# CT-Based Attenuation Correction Algorithm for Quantitative L-Shell X-Ray Fluorescence Imaging of Gold Nanoparticles in Murine Tumor Tissues

**DOI:** 10.3390/diseases13120403

**Published:** 2025-12-16

**Authors:** Marin Lohff, Gerret Haroske, Theresa Staufer, Jan Scheunemann, Florian Ziegler, Jannis Haak, Kazuya Kabayama, Xuhao Huang, Koichi Fukase, Florian Grüner

**Affiliations:** 1 Center for Free-Electron Laser Science (CFEL), University of Hamburg, Fachbereich Physik, Luruper Chaussee 149, 22761 Hamburg, Germany; 2 Department of Chemistry, Graduate School of Science, The University of Osaka, Toyonaka 560-0043, Osaka, Japan; 3 Interdisciplinary Research Center for Radiation Sciences, Institute for Radiation Sciences, The University of Osaka, Suita 565-0871, Osaka, Japan; 4 Forefront Research Center, Graduate School of Science, The University of Osaka, Toyonaka 560-0043, Osaka, Japan

**Keywords:** targeted radionuclide therapy, nanomedicine, X-ray fluorescence, XFI, gold nanoparticles, murine tumor sample

## Abstract

**Background:** Gold nanoparticles (GNPs) are widely used in nanomedicine as drug carriers, including in targeted radionuclide therapy where therapeutic radionuclides are bound to GNPs. Quantitative assessment of their biodistribution is essential. X-ray fluorescence imaging (XFI) is well suited for detecting high-Z elements, but its quantitative accuracy is compromised by strong attenuation effects, particularly in L-shell XFI where low-energy fluorescence (~10 to 12 keV) is heavily absorbed in tissue. **Methods:** We developed a computed tomography (CT)-guided attenuation correction algorithm for L-shell XFI. The method generates energy-dependent attenuation maps from co-registered CT data and performs voxel-wise corrections along both excitation and emission paths. The approach was tested on an ex vivo murine tumor sample resected three hours after intratumoral injection of 34.7 μg PEG-modified GNPs. **Results:** Application of the CT-guided correction substantially improved the relative accuracy of L-shell XFI reconstructions compared to uncorrected data. The corrected distribution maps showed consistent mass recovery across different measurement geometries, demonstrating that the algorithm compensates for the theoretically expected attenuation due to heterogeneous biological tissue. **Conclusions:** This study provides a proof-of-principle that CT-based attenuation correction enables more reliable and quantitative L-shell XFI of GNPs in biological samples. The approach represents a promising step toward accurate nanoparticle biodistribution assessment in biomedical research, including preclinical studies in targeted radionuclide therapy.

## 1. Introduction

Cancer remains a major global health and economic challenge, responsible for nearly one in six deaths worldwide [1]. In 2022, there were 20 million new cancer cases and 9.7 million deaths, and projections indicate that annual incidence may rise to 35 million by 2050 [1,2]. These numbers underscore the urgent need for innovative strategies for cancer diagnosis and therapy.

Nanomedicine has emerged as a promising avenue, offering platforms that combine diagnostic and therapeutic functions within a single agent, often termed (nano)theranostics [3]. Gold nanoparticles (GNPs) are among the most widely studied nanomaterials owing to their biocompatibility [4], surface functionalization versatility [5], and tunable optical and electronic properties [6]. These features enable their use as tumor-targeted contrast agents [7,8], where they can accumulate in tumors via both passive mechanisms such as the enhanced permeability and retention (EPR) effect [9] and active targeting through conjugated biomolecules [10,11,12]. In addition, GNPs can be functionalized to deliver therapeutic payloads. For example, Huang et al. recently demonstrated that ^211^At (astatine-211)-labeled GNPs combine the favorable surface chemistry of gold (Au) with the high potency of targeted alpha-particle therapy, achieving efficient radiolabeling, high stability, and promising therapeutic efficacy in preclinical cancer models [13]. Such examples illustrate the potential of GNPs as multifunctional theranostic agents.

Beyond radionuclide-based applications, recent advances in molecular imaging workflows increasingly rely on integrating anatomical and functional information through multimodal co-registration pipelines. For instance, atlas-based positron emission tomography/computed tomography (PET/CT) co-registration strategies have revealed that differences between registration output spaces can significantly affect biodistribution analysis in preclinical mouse models [14,15]. These findings underscore the importance of accurate anatomical priors when quantifying nanoparticle distributions, motivating the use of CT-derived information for X-ray-based elemental imaging approaches.

To realize this potential, non-invasive molecular imaging techniques are required to track nanoparticle biodistribution and pharmacokinetics with spatial and quantitative accuracy over extended time spans [16]. Conventional imaging modalities each face limitations for nanoparticle tracking. CT provides high spatial resolution (~0.5 mm) but requires millimolar contrast levels [17,18]. Magnetic resonance imaging (MRI) offers excellent soft-tissue contrast but poor molecular sensitivity (milli- to micromolar) and indirect, non-linear signal dependence [19,20]. Nuclear medicine techniques such as PET and single-photon emission computed tomography (SPECT) achieve nanomolar or even picomolar sensitivities but are constrained by limited spatial resolution (~3 mm) and the short half-lives of radiotracers [21,22,23]. A comparative overview of these techniques and their application to nanomedicine was provided by Arms et al. [24].

X-ray fluorescence imaging (XFI) offers a complementary alternative, providing elemental specificity, nanomolar sensitivity for gold concentrations [25], and the ability to simultaneously map multiple elements in tissue with micrometer-scale precision [26,27,28,29]. GNPs are particularly well suited for XFI because of gold’s high atomic number (Z=79), which yields strong X-ray interactions and stable signals independent of radioactive decay. This stability decouples injection from imaging, enabling longitudinal studies of nanoparticle retention, clearance, and tumor interactions.

X-ray fluorescence imaging has evolved considerably since its early in vivo applications in the 1970s, which were limited by strong attenuation of low-energy fluorescence and high Compton background. Early setups relying on radioactive sources and Ge(Li)-detectors suffered from insufficient sensitivity, but subsequent improvements, including the use of polarized excitation, optimized collimation, and better X-ray sources, reduced background and improved detectability. A major breakthrough was the emergence of synchrotron radiation in the 1980s, enabling highly focused, monochromatic, and polarized beams allowing for much higher sensitivities [29].

Although synchrotron access remains limited, recent advances in benchtop device based X-ray fluorescence computed tomography (XFCT)/XFI instrumentation and background-suppression algorithms have significantly improved practical applicability [30]. Furthermore, emerging compact X-ray sources, like laser-driven inverse Compton scattering systems offer bright, quasi-monochromatic beams and represent a promising technological pathway towards clinically deployable XFI systems [30,31].

XFI relies on detecting element-specific X-ray photons emitted following external excitation. L-shell XFI of GNPs offers distinct advantages over K-shell imaging, including a larger photoelectric cross-section and reduced scattering background at lower energies. These benefits, however, depend on sample size and excitation energy [32,33]. A major challenge for Au L-shell imaging is the substantial attenuation of low-energy fluorescence (~10 to 12 keV) within tissue, which leads to systematic underestimation of GNP concentrations [34,35]. And, if the sample size is too large, L-shell XFI becomes unpractical. However, the tissue sample sizes in our study were still small enough to allow for sufficient transmission of the photons. Attenuation affects both the incoming excitation beam and the emitted fluorescence photons, and depends on photon energy, tissue composition, and thickness.

Several strategies have been proposed to address this, including ratio-based methods using Compton scatter and Monte Carlo simulations [35,36]. While informative, these methods are limited in accuracy or practicality when applied to complex biological tissues. An alternative is to integrate anatomical information from co-registered imaging modalities such as CT, which can provide sample-specific attenuation maps [37]. Using CT-derived attenuation coefficients allows correction of both excitation and emission pathways on a voxel-wise basis.

Here, we present a CT-guided attenuation correction approach for L-shell XFI of GNPs in murine tissues. This proof-of-principle study evaluates whether co-registered CT information can improve recovery of GNP distributions compared to uncorrected XFI. Murine tumor and organ samples were imaged with synchrotron and compact laboratory XFI systems, reconstructed with and without correction, and the resulting GNP distributions quantitatively compared. By addressing the fundamental challenge of fluorescence attenuation, this work represents a step toward more reliable, quantitative XFI for nanoparticle biodistribution studies. As only a single tumor sample was analyzed, this work should be regarded as a methodological validation study rather than a biological or pharmacokinetic investigation.

## 2. Materials and Methods

### 2.1. Murine Sample Preparation

The experimental protocol was approved by the Animal Care and Use Committee of the Osaka University Graduate School of Science (approval code: 2019-02-1, approval date: 1 April 2019, validity period: five years). BALB/c-nu/nu mice (Japan SLC Inc.; Hamamatsu, Japan) were maintained under pathogen-free conditions with free access to food and water. To establish subcutaneous tumors, $1\times 10^{7}$ human pancreatic tumor cells (PANC-1) were injected into the hind leg of two mice. Tumor growth was monitored, and animals were included for imaging studies once tumors reached a volume of approximately 50 mm^3^. Each mouse then received a single intratumoral injection of PEG-modified gold nanoparticles (GNP@mPEG) corresponding to a theoretical dose of 34.7 μg Au. This amount is typical for such preclinical studies towards radionuclide therapy.

Three hours after injection, the animals were euthanized, and the tumor, spleen, and liver were excised. Excised tissues were embedded in paraffin for later imaging experiments. Because the tumors were located on the hind legs, one resected tumor sample also contained adjacent femur and pelvic bone. Although unintentional, this heterogeneity provided a useful test case for evaluating attenuation effects and validating the correction algorithm. This tumor sample measured approximately 19×16×12 mm. The other tumor sample (not reported here) was of similar size.

### 2.2. Nanoparticle Preparation

Gold nanoparticles with a nominal core diameter of 5 nm (OD 1, stabilized in citrate buffer; Sigma-Aldrich, St. Louis, MO, USA) were used in this study. For surface modification, methoxy-polyethylene glycol thiol (mPEG-SH, Mn 6000) was added to the GNP suspension to a final concentration of 0.1 mol/L. The reaction mixture was stirred at room temperature for 2 h, after which PEG-modified GNPs (GNP@mPEG) were purified by centrifugation as described previously in [13].

The quality of the PEGylated GNPs was confirmed in this earlier study by transmission electron microscopy (JEM-2100; JEOL Ltd., Tokyo, Japan), dynamic light scattering (DLS), zeta potential analysis (Zetasizer Ultra; Malvern Panalytical Ltd., Malvern, UK), and UV–Vis spectroscopy (V-730; JASCO Corp., Tokyo, Japan). For the present experiments, only 5 nm GNP@mPEG were used.

### 2.3. Measurements

XFI scans were performed both at the P21.1 beamline of the PETRA III synchrotron (DESY, Hamburg, Germany) and with a compact laboratory XFI system that is currently under development by the University of Hamburg (UHH) in the framework of a project funded by the German Federal Ministry of Research, Technology and Space (BMFTR). All crucial parameters and measurements taken are listed in Table 1.

To enable 3D reconstruction of the GNP distribution, the tumor sample was scanned from four orientations: front F, back B, left L, and right R. At each raster scan point, fluorescence spectra were recorded using silicon drift detectors (XR-100 FAST SDD; Amptek Inc., Bedford, MA, USA). Each detector featured a 1 mm chip thickness, a collimated active area of 50 mm^2^, and a 12.5 μm thick beryllium entrance window.

Complementary high-resolution CT scans were acquired with a preclinical scanner (SmART+; Precision X-Ray, North Branford, CT, USA) to provide anatomical data for attenuation correction. Acquisition parameters were: tube voltage 40 kVp, tube current 8 mA, scan duration 1 min, and voxel resolution 0.1 mm.

### 2.4. CT-Based Attenuation Correction Algorithm

A numerical algorithm was developed to correct for photon attenuation using the CT-derived anatomical information. The workflow was as follows:

1. Voxel model: The CT dataset was converted into a 3D voxel model, with each voxel assigned a Hounsfield Unit (HU).
2. Material mapping: Based on predefined HU ranges, voxels were classified into material types (air, paraffin wax, adipose tissue, soft tissue, cortical bone), yielding a sample-specific 3D material map. Note that tumor tissue is here approximated as adipose and soft tissue. This assumes the change in the tumor tissue’s elemental composition and elemental weight fractions during progression until the resection is negligible. Also, there is no NIST compound catalog entry for this type of tissue.
  The HU thresholds were initially derived from literature values (e.g., [38]), which correlate CT numbers with physical tissue parameters. These values were then iteratively refined by manual inspection of the CT slices, ensuring accurate anatomical segmentation of the specific samples. The defined HU ranges are listed in Table 2.
3. Path-dependent attenuation: For each scan point and detector, attenuation of both the incident beam and the outgoing fluorescence photons was computed using the Beer–Lambert law [39]:
  (1) $$T=\frac{N}{N_{0}}=\exp\left(-\left(\frac{\mu }{\rho }\right)\int \rho \;dx\right),$$
  where μ/ρ denotes the energy- and material-specific mass attenuation coefficient, obtained from the xraylib database [40]. Transmission factors *T* were calculated separately along all excitation and emission paths yielding a mean attenuation value for each scan position and detector.
4. Correction factor: For each scan point, the inverse of the combined transmission factor, averaged over all potential fluorescence origins and weighted by detector geometry and efficiency, yielded the final correction factor κ.

Further mathematical details of the attenuation correction are provided in Appendix A.

### 2.5. Data Analysis

To determine the number of detected fluorescence photons at each scan point, the measured fluorescence spectrum was fitted. A model consisting of multiple Gaussian functions, one for each characteristic fluorescence line of the elements of interest, was fitted to the data on top of a composite background function. This background model consisted of a constant offset and the sum of exponential, polynomial, and sigmoidal terms. The exponential term accounts for the steep decrease in counts towards higher energies, the polynomial term provides flexibility for the smooth continuum, and the sigmoidal component models the low-energy shoulder that arises from detector response and scattered radiation. This composite approach provided a stable and unbiased fit over the selected 8 to 15 keV range. The peak widths were constrained to detector resolution values (123 eV FWHM resolution at 5.9 keV), while the peak positions were fixed at tabulated fluorescence energies from the xraylib database. The fitting procedure is described in detail by Körnig et al. [41]. The goodness of fit was evaluated using the reduced chi-squared ($\chi^{2}$) value, which is a measure of the discrepancy between the observed data and the fitted model [42]. A reduced $\chi^{2}$-value close to 1 indicates a good fit, suggesting that the model accurately describes the data.

To ensure accurate quantification and to prevent the misallocation of counts, it is crucial to include multiple fluorescence peaks for each element of interest and of the elements present in the setup. This is particularly important for the Au L-fluorescence lines, which are in close proximity to other fluorescence lines, like tungsten (W) and tantalum (Ta). The presence of various elements in the sample and experimental setup can be attributed to several sources. Tungsten fluorescence originates in case of the synchrotron primarily from upstream components and in case of the compact laboratory system from the X-ray tube’s anode, while Ta is used in the absorbers plates, which are used to attenuate the synchrotron beam. The tungsten fluorescence signal is predominantly observed in the compact laboratory system, whereas it is not apparent in the beamline setup. Strontium (Sr) is a naturally occurring trace element in bone material [43]. Nickel (Ni) is found in the detector cover of the silicon drift detectors (SDDs), and rubidium (Rb) and zinc (Zn) are present as trace elements in the biological tissue [44,45,46]. To allocate the correct number of counts to Au, a broader fit range from 8 to 15 keV was found to be optimal. This includes multiple fluorescence lines from abundant elements within the vicinity of the Au fluorescence.

**Figure 1:**
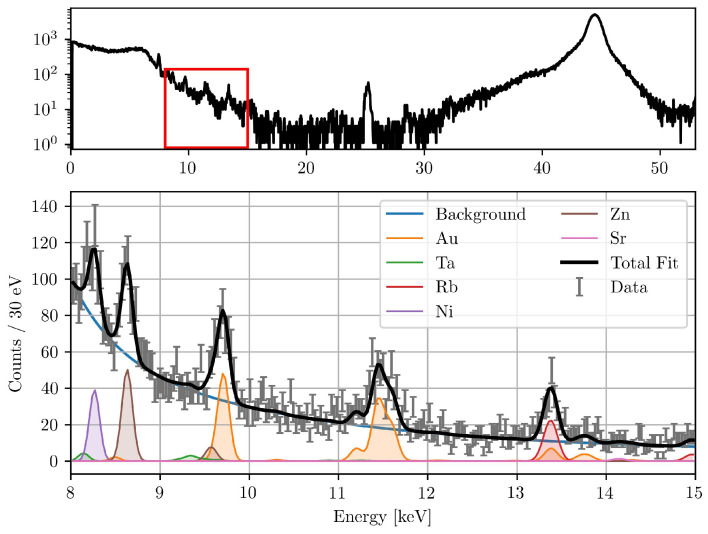
Typical XFI spectrum (**top**) during the beamline measurements. The fitted spectrum of the red marked region is also shown (**bottom**). The detector was positioned at 150° to the excitation beam. Visible are the fitted peaks of different elements present in the setup, the background function, and the total fit with a reduced $\chi^{2}$-value of 1.33.

The total corrected fluorescence counts were then calculated by multiplying the fitted counts by the correction factor κ. A representative measured and fitted spectrum, showing the deconvolution of elemental peaks, is shown in Figure 1.

The mass of the element of interest can be determined by

(2) $$m=\frac{N_{\mathrm{corr}}\;\cdot \;A_{\mathrm{Beam}}}{N_{0}\;\cdot \;\sigma_{f}},$$

with *m* being the elemental mass (in g) contained within the irradiated beam volume at scan position (x,y). $N_{0}$ is the number of incident photons, $A_{\mathrm{Beam}}$ the cross-sectional area of the beam and $\sigma_{f}$ the fluorescence cross-section (in $cm^{2}/g$), which is retrieved from the xraylib database [40]. The correct number of fluorescence photons $N_{\mathrm{corr}}$ stems from the number of detected fluorescence photons *N* multiplied by the correction factor κ.

The statistical uncertainty in *m* is primarily governed by the counting statistics of *N*, propagated through the fitting error. Systematic uncertainties arise from the attenuation correction factor κ and the fluorescence cross-section $\sigma_{f}$.

## 3. Results

**Figure 2:**
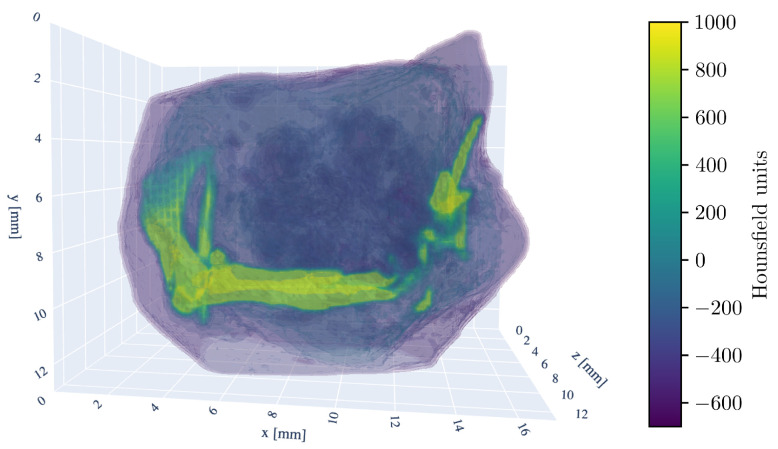
Three-dimensional rendering of the tumor sample CT-scan. The HU window is set from −700 to 1000 HU. Visible are the murine femur bone in the center, the pelvic bone (ilium) on the left side, and fragments of the fibula and tibia on the right. The kneecap (patella) is also discernible in the bottom right.

For a three-dimensional understanding of the tumor sample, the 3D model of the CT scan is shown in Figure 2.

For the liver and spleen samples, the Au signal did not exceed the significance threshold. Therefore, they are precluded from further quantitative evaluation.

### 3.1. Quantitative Impact of Attenuation Correction

**Figure 3:**
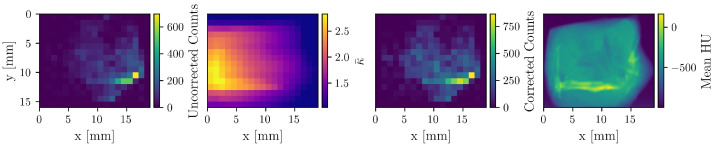
The first three maps correspond to the detector at −90°, which is situated on the right side in this visualization. The uncorrected count, correction factor, and corrected count maps of Au are shown. The fourth map shows the mean HU-values of the CT image. Apparent is the enhanced correction on the left side of the sample, as well as the attenuation “shadow” of the bone.

To demonstrate the impact of the attenuation correction, Figure 3 displays an uncorrected count map, the corresponding correction factor (κ) map, the resulting corrected count map, and the mean HU-value map derived from the CT scan. A reconstruction based on the uncorrected count map yields a total Au mass of 6 μg, whereas attenuation correction increases the value to 11.3 μg, underscoring the necessity of this procedure for quantitative accuracy.

### 3.2. Composite X-Ray Fluorescence and CT Imaging

**Figure 4:**
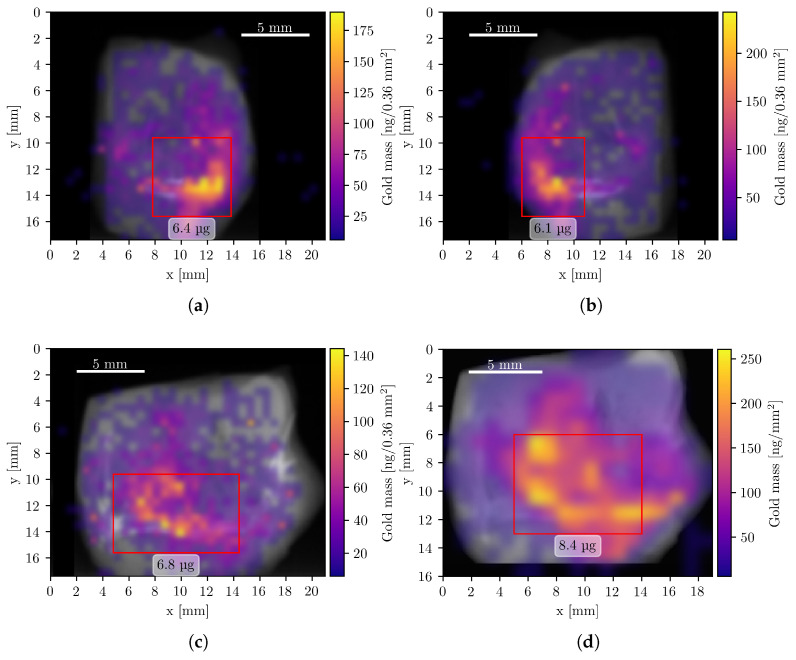
Composite Au XFI/CT images of the tumor sample measurements. All pixels with a Au mass value below 6 ng are fully transparent. Data from panels (**a**–**c**) were acquired with the compact system, (**d**) during the beamline measurement. Red rectangles show regions of interest with a high concentration of Au and the corresponding mass within this region. (**a**) Left orientation with a total Au mass of 14.5 μg. (**b**) Right orientation with a total Au mass of 14.7 μg. (**c**) Back orientation with a total Au mass of 14.5 μg. (**d**) Front orientation (mirrored) with a total Au mass of 15.8 μg.

To visualize the spatial distribution of GNPs, composite images were generated by superimposing the elemental mass maps obtained from XFI onto summed CT projection images, which resemble conventional radiographs. The XFI mass maps were smoothed using Gaussian interpolation, corrected for attenuation, and expressed in units of mass per pixel. Figure 4 shows the resulting composite images of the Au L-line fluorescence, representing the GNP distribution in four measurement orientations.

A minor alignment discrepancy is visible between the compact scan in orientation B (Figure 4c) and the beamline scan (Figure 4d), which is attributed to the different experimental setups and sample mounting procedures.

### 3.3. Strontium as an Internal Fiducial Marker

**Figure 5:**
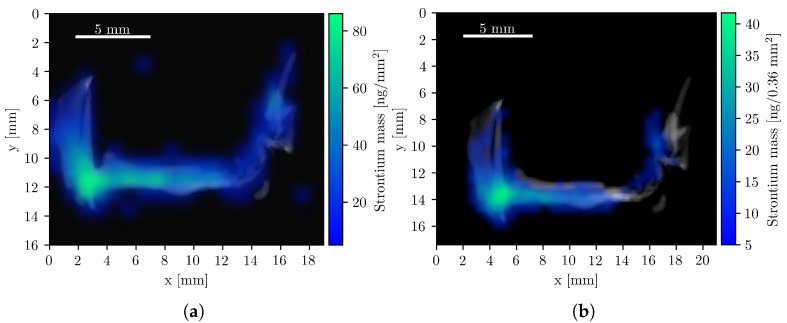
Composite Sr XFI/CT images with the isolated bone structure. All pixels with a Sr mass value below 5 ng are fully transparent. (**a**) Beamline measurement with a total Sr mass of 2.4 μg. (**b**) Compact system measurements with a total Sr mass of 2.6 μg.

Strontium (Sr) composite images were also generated, as Sr is naturally abundant in bone tissue. Its colocalization with bone provides an internal fiducial marker that can aid in the co-registration of CT and XFI datasets, a role typically filled by external reference objects [47]. To this end, the bone structure was first computationally isolated from the CT image, and the Sr K-line mass maps from both the compact system and the beamline setup were overlaid, as shown in Figure 5. The quantified Sr mass was 2.6 μg and 2.4 μg, respectively. The concentration of Sr is highest in the femoral head and pelvic joint, consistent with regions of elevated bone remodeling due to mechanical load [43,48].

### 3.4. Elemental Correlation Analysis

**Figure 6:**
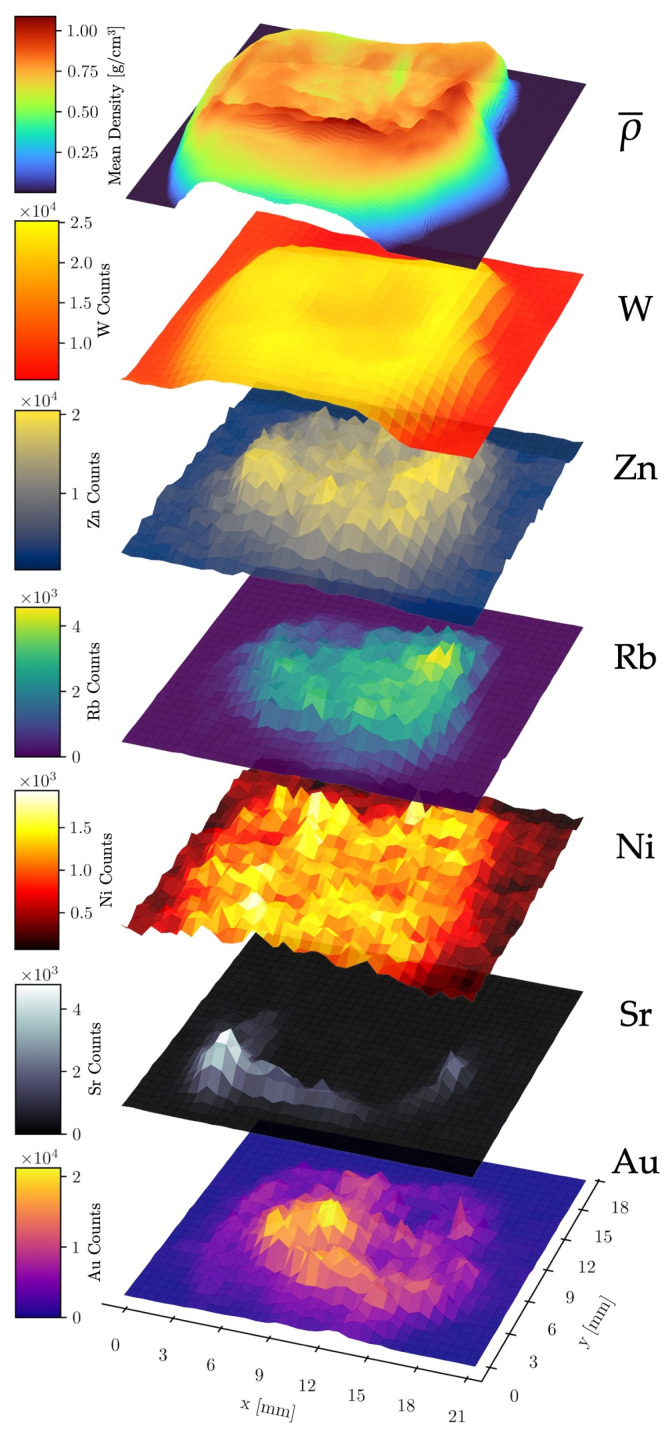
Stacked surface plots of elemental count distributions of Au, Sr, Ni, Rb, Zn, and W. The data was acquired with the compact laboratory system. The top layer shows the mean density along the *z*-axis through the sample.

To further investigate possible inter-element and element–density relationships, stacked surface plots were created to illustrate the spatial distribution of the mean density $\bar{\rho }$ (along the *z*-axis, see Figure 2) and the relative abundance of six different elements across the raster scan area (Figure 6). This analysis utilized the combined data from all four detectors of the compact system measurement.

No significant correlation was observed between W and Au counts, validating the effectiveness of the spectral fitting routine in distinguishing these elements despite the partial overlap of their fluorescence lines. A strong positive correlation was found between Sr counts and the mean density, as expected, since Sr is localized within the dense bone matrix. A second correlation was observed between W counts and the mean density. As the W fluorescence originates primarily from upstream components such as apertures and optics (the W fluorescence of the X-ray tube’s anode is partly filtered out), its intensity serves as a proxy for X-ray scattering, which scales with the mean density along the beam path. The nearly constant W signal in sample-free areas is likely due to fluorescence of the detector itself, scattering from air and fluorescence from shielding materials. Other abundant elements in the sample (Sr, Au, Rb, Zn, Ni) also correlated with the mean density. However, the Ni signal exhibited a significantly higher noise level in sample-free regions, likely caused by fluorescence from the nickel used in the detector housing of the SDDs [49].

These findings confirm that the spectral fitting and attenuation correction reliably distinguish GNP fluorescence from other sources, providing a solid basis for quantitative biodistribution analysis.

### 3.5. GNP Biodistribution Coefficients

The nanoparticle biodistribution coefficients (NBCs), which measure the concentration of an injected nanoparticle type in biological tissue, were calculated as the percentage of injected dose per gram of tissue (%ID/g) [50]. This metric is defined as

(3) $$\%\mathrm{ID}/g=\frac{m_{Au}}{34.7\;\mu g}\;\cdot \;\frac{1}{m_{\mathrm{tissue}}}\;\cdot \;100,$$

where $m_{Au}$ is the quantified Au mass and $m_{\mathrm{tissue}}$ is the tissue mass. For liver and spleen, the Au signal did not exceed the significance threshold, indicating minimal accumulation in these organs after intratumoral injection. They were therefore excluded from further consideration.

The resulting nanoparticle biodistribution coefficients (NBCs) show that the tumor retained 42.9 %ID, corresponding to a concentration of 26.8 %ID/g. The average reconstructed gold mass within the tumor was 14.9 μg.

Important to note is that the calculation is based on a single tumor sample, which precludes statistical analysis. Furthermore, the injected dose was localized, so the NBC values do not generalize to systemic administration. Despite these caveats, the results provide an initial quantitative reference point for imaging intratumorally administered GNPs with XFI.

## 4. Discussion

This proof-of-principle study demonstrates that a CT-based numerical algorithm provides a consistent correction for the theoretically expected attenuation from biological tissue in L-shell XFI, enabling quantitative analysis of GNP distributions in ex vivo samples. The accuracy of this quantification hinges on several factors, including the precise segmentation of the CT data into different material types and the accuracy of the mass attenuation coefficients used in the calculations. Here, the segmentation of CT voxels into five material classes (air, paraffin, adipose tissue, soft tissue, bone) reflects the composition of the paraffin-embedded murine hindleg and is therefore sufficient for this sample type. However, more heterogeneous or anatomically complex samples would require more refined segmentation strategies to avoid bias in the attenuation correction. Future work could include the use of dual-energy CT to improve material differentiation and reduce uncertainties in the attenuation correction.

One of the most significant findings from the data was the highly inhomogeneous distribution of GNPs within the tumor sample. The mass reconstructions from all four measurements (acquired using two different systems) yielded total mass values that were in close agreement. This highlights the robustness of the algorithm’s correction, since the sample exhibits varying attenuation depending on its orientation.

The use of endogenous strontium as a fiducial marker for image co-registration proved highly effective. The strong correspondence between the strontium fluorescence signal and bony structures in the CT images enabled accurate alignment of both modalities without the need for implanted markers, which can be invasive or impractical [47,51,52]. Leveraging naturally occurring elements for registration may therefore broaden the applicability of multimodal imaging workflows in preclinical research.

These results also fit within broader developments in quantitative nanoparticle imaging. In related work on atlas-based segmentation of PET images in mouse models [15], it has been demonstrated that the choice of the registration space significantly impacts radiomics analysis and biodistribution assessment. The current XFI method provides an orthogonal approach to functional imaging modalities like PET, SPECT, or CT by offering element-specific information that is particularly valuable for tracking metallic nanoparticles or molecular tracers such as iodine. Moreover, XFI can simultaneously detect multiple elements to provide both functional and anatomical information, for instance by visualizing the endogenous strontium distribution in bone tissue. A recent study shows that metallic nanoparticles, like palladium-doped systems, can be tracked using integrated workflows combining XFI, Inductively Coupled Plasma Mass Spectrometry (ICP-MS), and imaging mass cytometry. In the approach presented in [53], XFI provides non-destructive, element-specific maps with higher sensitivity than CT, ICP-MS provides absolute quantification, and mass cytometry gives cellular-scale resolution. Our CT-based attenuation correction naturally complements such hierarchical workflows by providing anatomically accurate and quantitatively reliable XFI maps without additional sample preparation.

The ability to directly detect therapeutic nanoparticles without radiolabeling and without decay-related signal loss is particularly advantageous for long-term biodistribution studies and aligns with the increasing emphasis on translational theranostic strategies, like in recent work on theranostic approaches for gastric cancer [14]. Accurate co-registration using endogenous markers, combined with anatomical attenuation correction, positions this method as a useful complement to PET/CT and other preclinical modalities, especially when tracking high-Z agents. As nanoparticle-mediated therapies continue to advance, robust quantification of their distribution will remain essential for validating targeting efficiency and informing dosimetry [54,55,56,57,58,59].

The primary limitations of the technique remain the shallow penetration depth of Au L-shell fluorescence photons, which restricts its application to smaller samples or superficial targets and the long scan times (approximately 2.5 h for one beamline measurement and 11.5 h for one measurement with the compact XFI system) required to achieve sufficient statistics. Furthermore, the compact laboratory XFI system used in this study is still under development with two prototype demonstrators, but it is not yet available for routine measurements.

Advances in detector technology, higher-flux X-ray sources, and data acquisition strategies, such as fly-scanning techniques, could help to reduce the required measurement time and enable in vivo application and clinical translation of XFI [30,31,60,61,62]. Despite progress in background-reduction algorithms, Compton scatter background continues to impose a practical limit on XFI, restricting its application to small or superficial samples [63]. However, the high sensitivity, with a (theoretical) detection limit of merely 0.6 μg of Au, demonstrates its potential for preclinical research, where high sensitivity is often crucial for detecting small amounts of tracer material.

## 5. Conclusions

In conclusion, this work has successfully demonstrated the feasibility of using a CT-based attenuation correction algorithm for quantitative L-shell XFI of gold nanoparticles in ex vivo tissue samples. The study highlights the importance of accurate attenuation correction for obtaining reliable quantitative results and reveals the highly inhomogeneous distribution of intratumorally injected GNPs. Furthermore, the use of endogenous strontium as a fiducial marker for image co-registration represents a novel and valuable approach for multimodal imaging studies. While the technique has limitations in terms of penetration depth and scan time, its high sensitivity and quantitative capabilities make it a promising tool for preclinical research, particularly for studies investigating the biodistribution and therapeutic efficacy of high-Z nanoparticles. Future developments should focus on improving the accuracy of the attenuation correction, reducing the scan time and extending the applicability of this technique to in vivo imaging. Note that these findings about the attenuation correction could also be applied to K-shell XFI in cases where the excitiation energy is sufficient. However, the attenuation correction for the lower energy of L-shell is more demanding and hence we have chosen this case.

## Figures and Tables

**Table 1:** Summary of scan parameters and sample orientations.

	P21.1 Beamline	Compact XFI System
Scanned samples/ orientation	Tumor F Liver Spleen	Tumor LRB
Beam energy (keV)	53	59
Bandwidth E/ΔE	$10^{-3}$	$10^{-1}$
Photon flux ^1^ (ph/s)	$4.79\times 10^{8}$	$3.84\times 10^{8}$
Beam cross-section ^2^ (mm^2^)	1×1	0.65×0.6 (rms)
Number of detectors	10	4
Detector distance (cm)	6	4
Raster scan step size (mm)	1	0.6
Acquisition time (s/point)	30	40

^1^ The photon flux value of the synchrotron is due to the use of tantalum (Ta) absorber plates to keep the detector’s input count rate small enough. ^2^ The compact source had a Gaussian profile, whereas the synchrotron beam was square shaped.

**Table 2:** HU-regions used to define the material type in the voxel-model.

HU Range	NIST Compound
−1000 to −750	Air, dry (near sea level)
−750 to −350	Paraffin wax
−350 to −100	Adipose tissue (ICRP)
−100 to 250	Tissue, soft (ICRP)
250 to 2000	Bone, cortical (ICRP)

## Data Availability

The datasets used and/or analyzed during the current study are available from the corresponding author upon reasonable request.

## Abbreviations

The following abbreviations are used in this manuscript:

XFI	X-ray fluorescence imaging
GNP	Gold nanoparticle
PEG	Polyethylene glycol
CT	Computed tomography
EPR	Enhanced permeability and retention
MRI	Magnetic resonance imaging
PET	Positron emission tomography
SPECT	Single-photon emission computed tomography
Z	Atomic number
XFCT	X-ray fluorescence computed tomography
PETRA	Positron–electron tandem ring accelerator
DESY	Deutsches Elektronen-Synchrotron
HU	Hounsfield unit
NIST	National Institute of Standards and Technology
ICRP	International Commission on Radiological Protection
FWHM	Full width at half maximum
SDD	Silicon drift detector
NBC	Nanoparticle biodistribution coefficient
ID	Injected dose
ICP-MS	Inductively coupled plasma mass spectrometry

## Appendix A. Mathematical Formulation of the Attenuation Correction

The sample is represented in a Cartesian coordinate system with a position vector r=(x,y,z). The incident beam is assumed to propagate along the *z*-axis, and the detector is placed at a fixed point $r_{\det}$. The primary photon energy of the excitation beam is denoted as $E_{0}$, while the energy of the fluorescence photons of interest is $E_{f}$.

At any position r, the sample has a local volumetric mass density ρ(r) in $g/cm^{3}$. As a first step, the CT dataset was converted from Hounsfield units (HU) into a three-dimensional mass density map. This conversion is required because the mass attenuation coefficients used in the transmission calculations (A1) depend directly on the local mass density. The calibration curve was derived from a reference measurement of water and air. An additional reference point was fixed at 2000 HU for bone. A piecewise linear regression was then done between HU and ρ by fitting air–water and water–bone intervals separately (for the scope of attenuation correction in this setting a bilinear calibration curve is adequate and provides sufficiently accurate density values). The densities for air, water, and bone were set to 0.0012, 1.0, and 1.85 g/cm^3^, respectively. Densities for all voxels were obtained by applying this mapping, with voxels segmented as air explicitly fixed to $\rho_{\mathrm{air}}$ to suppress noise outside the sample. The linear regression parameters were further refined such that the integrated density of the reconstructed volume agreed with the known bulk mass of the sample. This adjustment guaranteed consistency between the voxelized model and the physical sample.

The resulting density distribution ρ(r) provided the basis for calculating the linear attenuation coefficient as

(A1) $$\mu \left(r,E\right)=\left(\frac{\mu }{\rho }\right)\left(r,E\right)\;\rho \left(r\right),$$

with units 1/cm, which quantifies the probability per unit path length that a photon of energy *E* is absorbed or scattered. The incoming beam delivers $N_{0}$ photons during the scan dwell time. Finally, $\sigma_{f}\left(E_{0}\right)$ denotes the effective fluorescence production cross-section per gram of tracer material ($cm^{2}/g$). This quantity combines the absorption cross-section for the relevant atomic shell, the fluorescence yield, and the emission probability of the specific fluorescence line. The Beer–Lambert law states, that the intensity of a monochromatic beam decreases exponentially with depth due to absorption and scattering processes. Each infinitesimal thickness ds attenuates a fraction μ(r,E)ds of the beam. Integration over the entire path gives

(A2) $$T\left(L;E\right)=\exp\;\left(-\int_{L}\mu \left(r,E\right)\;ds\right),$$

which is the total transmission *T* of the beam after traversing path L.

The local incident flux at point r is reduced both by external beam filters, expressed by a factor $T_{\mathrm{filters}}\left(E_{0}\right)$, and by absorption along the incoming path $L_{\mathrm{in}}\left(r\right)$ from the sample surface to r. It is therefore given by

(A3) $$N\left(E_{0},r\right)=N_{0}\;T_{\mathrm{filters}}\left(E_{0}\right)\;\exp\;\left(-\int_{L_{\mathrm{in}}\left(r\right)}\mu \left(r^{\prime },E_{0}\right)\;ds^{\prime }\right).$$

At that location, fluorescence photons are generated proportionally to the incident flux, the tracer density $\rho_{Au}\left(r\right)$, and the fluorescence cross section. The differential production rate per path length is

(A4) $$\frac{dN_{f}}{ds}\left(r\right)=\sigma_{f}\left(E_{0}\right)\;N\left(E_{0},r\right)\;\rho_{Au}\left(r\right).$$

Integrating along the beam path yields the total number of fluorescence photons emitted:

(A5) $$N_{f}^{\mathrm{emitted}}=\int_{L_{\mathrm{in}}}\sigma_{f}\left(E_{0}\right)\;N\left(E_{0},r\right)\;\rho_{Au}\left(r\right)\;ds.$$

Fluorescence photons themselves are attenuated when traveling to the detector. The survival probability (transmission) from a point r to the detector is

(A6) $$T_{\mathrm{out}}\left(r;E_{f}\right)=\exp\;\left(-\int_{L_{\mathrm{out}}\left(r\right)}\mu \left(r^{\prime },E_{f}\right)\;ds^{\prime }\right).$$

Additional multiplicative factors account for attenuation in air, in beryllium windows, and in the detector itself, as well as geometric collection efficiency. The overall detection probability is therefore

(A7) $$P_{\det}\left(r;E_{f}\right)=\varepsilon_{\mathrm{geo}}\left(r\right)\;T_{\mathrm{out}}\left(r;E_{f}\right)\;T_{\mathrm{air}}\left(E_{f}\right)\;T_{\mathrm{Be}}\left(E_{f}\right)\;\varepsilon_{\mathrm{int}}\left(E_{f}\right),$$

where the geometric efficiency

(A8) $$\varepsilon_{\mathrm{geo}}\left(r\right)\approx \frac{A_{\det}}{4\pi \;d{\left(r\right)}^{2}}$$

follows from the detector solid angle subtended at r, with $A_{\det}$ the sensitive detector area and d(r) the source–detector distance. The intrinsic efficiency

(A9) $$\varepsilon_{\mathrm{int}}\left(E\right)=1-\exp\;\left(-{\frac{\mu }{\rho }}_{\det}\left(E\right)\;\rho_{\det}\;t_{\mathrm{chip}}\right)$$

is the absorption probability in the detector material, with $\rho_{\det}$ and $t_{\mathrm{chip}}$ its density and thickness.

Combining production and detection, the line integral for the detected counts is

(A10) $$N_{f,\det}=\int_{L_{\mathrm{in}}}\sigma_{f}\left(E_{0}\right)\;N\left(E_{0},r\right)\;\rho_{Au}\left(r\right)\;P_{\det}\left(r;E_{f}\right)\;ds.$$

In practice the sample is discretized into voxels. When the excitation beam and fluorescence photons traverse the voxelized geometry, the exact intersection lengths of the beam and fluorescence photon within each voxel is calculated using a line–voxel intersection routine. This yields a sequence S of voxels indexed by *j*, each associated with its material label, density, and the precise path length $\ell_{j}$ that the beam or fluorescence photon spends inside that voxel. With these lengths, the path integrals can be expressed as discrete sums:

(A11) $$\begin{array}{cc} \int_{L}\mu \left(r,E\right)\;ds & \approx \sum_{j\in S}{\left(\frac{\mu }{\rho }\right)}_{j}\left(E\right)\;\rho_{j}\;\ell_{j}, \end{array}$$

(A12) $$\begin{array}{cc} N_{f,\det} & \approx \sum_{j\in S}\sigma_{f}\left(E_{0}\right)\;N\left(E_{0},r_{j}\right)\;\rho_{Au,j}\;P_{\det}\left(r_{j};E_{f}\right)\;\ell_{j}. \end{array}$$

Here $\rho_{j}$ is the mass density of voxel *j*, $\rho_{Au,j}$ the tracer density, and $r_{j}$ the voxel center.

For practical correction, one introduces multiplicative factors. The attenuation factor $\kappa_{j}$ accounts for incoming and outgoing transmission through sample, air and windows:

(A13) $$\frac{1}{\kappa_{j}}=T_{\mathrm{filters}}\left(E_{0}\right)\;T_{\mathrm{in},j}\left(E_{0}\right)\;T_{\mathrm{out},j}\left(E_{f}\right)\;T_{\mathrm{air}}\left(E_{f}\right)\;T_{\mathrm{Be}}\left(E_{f}\right),$$

while the efficiency factor $\xi_{j}$ collects geometry and detector absorption:

(A14) $$\frac{1}{\xi_{j}}=\varepsilon_{\mathrm{geo}}\left(r_{j}\right)\;\varepsilon_{\mathrm{int}}\left(E_{f}\right).$$

With these definitions, the voxel contribution becomes

(A15) $$\Delta N_{f,\det}^{\left(j\right)}=\sigma_{f}\left(E_{0}\right)\;N_{0}\;\rho_{Au,j}\;\ell_{j}\;\cdot \;\frac{1}{\kappa_{j}}\frac{1}{\xi_{j}}.$$

Summing over all voxels within the excitation beam, the corrected counts are

(A16) $$N_{\mathrm{corr}}=\sum_{j\in S}\kappa_{j}\xi_{j}\;\Delta N_{f,\det}^{\left(j\right)}=\sigma_{f}\left(E_{0}\right)\;N_{0}\sum_{j\in S}\rho_{Au,j}\;\ell_{j}.$$

The right-hand expression shows that, after correction, the counts are directly proportional to the sum of the tracer densities in voxels of sequence S. Multiplying by the beam cross-sectional area $A_{\mathrm{beam}}$ gives the tracer mass in the illuminated column:

(A17) $$m_{Au}=\frac{N_{\mathrm{corr}}\;A_{\mathrm{beam}}}{\sigma_{f}\left(E_{0}\right)\;N_{0}}.$$

Experimentally, the spectrum recorded at the detector is fitted with a model consisting of a background $f_{B}\left(E;c_{B}\right)$ and a sum of Gaussian peaks for the fluorescence lines:

(A18) $$f\left(E;c_{B}\right)=f_{B}\left(E;c_{B}\right)+\sum_{i}\sum_{j}A^{i,j}\exp\;\left(-\frac{{\left(E-E_{f}^{i,j}\right)}^{2}}{2\;\delta E^{2}}\right).$$

Here $A^{i,j}$ is the fitted amplitude for line (i,j) and δE is the detector resolution. The integrated counts in a peak are

(A19) $$N_{f,\det}^{i,j}=A^{i,j}\;\delta E\sqrt{2\pi }.$$

These measured values are then corrected with the κ and ξ factors, that are averaged over the excitation voxels, yielding $N_{\mathrm{corr}}$ which directly recovers the tracer mass according to the relation above.
